# Efficacy of a Community-Based Physical Activity Program KM2H^2^ for Stroke and Heart Attack Prevention among Senior Hypertensive Patients: A Cluster Randomized Controlled Phase-II Trial

**DOI:** 10.1371/journal.pone.0139442

**Published:** 2015-10-01

**Authors:** Jie Gong, Xinguang Chen, Sijian Li

**Affiliations:** 1 Department of Chronic Diseases, Wuhan Center for Disease Prevention and Control, Wuhan, China; 2 Department of Epidemiology, University of Florida, Gainesville, Florida, United States of America; 3 School of Nursing, Hong Kong Polytechnic University, Hong Kong, China; University of Glasgow, UNITED KINGDOM

## Abstract

**Objective:**

To evaluate the efficacy of the program *Keep Moving toward Healthy Heart and Healthy Brain* (KM2H^2^) in encouraging physical activities for the prevention of heart attack and stroke among hypertensive patients enrolled in the Community-Based Hypertension Control Program (CBHCP).

**Design:**

Cluster randomized controlled trial with three waves of longitudinal assessments at baseline, 3 and 6 months post intervention.

**Setting:**

Community-based and patient-centered self-care for behavioral intervention in urban settings of China.

**Participants:**

A total of 450 participants diagnosed with hypertension from 12 community health centers in Wuhan, China were recruited, and were randomly assigned by center to receive either KM2H^2^ plus standard CBHCP care (6 centers and 232 patients) or the standard care only (6 centers and 218 patients).

**Intervention:**

KM2H^2^ is a behavioral intervention guided by the Transtheoretical Model, the Model of Personalized Medicine and Social Capital Theory. It consists of six intervention sessions and two booster sessions engineered in a progressive manner. The purpose is to motivate and maintain physical activities for the prevention of heart attack and stroke.

**Outcome Measures:**

Heart attack and stroke (clinically diagnosed, primary outcome), blood pressure (measured, secondary outcome), and physical activity (self-report, tertiary outcome) were assessed at the individual level during the baseline, 3- and 6-month post-intervention.

**Results:**

Relative to the standard care, receiving KM2H^2^ was associated with significant reductions in the incidence of heart attack (3.60% vs. 7.03%, p < .05) and stroke (5.11% vs. 9.90%, p<0.05), and moderate reduction in blood pressure (-3.72mmHg in DBP and -2.92 mmHg in DBP) at 6-month post-intervention; and significant increases in physical activity at 3- (*d* = 0.53, 95% CI: 0.21, 0.85) and 6-month (*d* = 0.45, 95% CI: 0.04, 0.85) post-intervention, respectively.

**Conclusion:**

The program KM2H^2^ is efficacious to reduce the risk of heart attack and stroke among senior patients who are on anti-hypertensive medication. Findings of this study provide solid data supporting a formal phase-III trial to establish the effectiveness of KM2H^2^ for use in community settings for prevention.

**Trial Registration:**

ISRCTN Register ISRCTN12608966

## Introduction

The prevalence of hypertension in China was approximately 5% in the 1950s [1]. It took four decades for the rate to reach 10% in the 1990s [2,3]. However, this rate was tripled in 20 years and reached 30.1% in 2011, along with the rapid economic growth in China [3]. More alarming than the rapid increases in the prevalence of hypertension is the large number of senior hypertensive patients. Consistent with results from different sources [4–6], findings from a national random survey conducted in 2009–10 indicated that 58.2% (95% CI: 56.5, 59.9) of Chinese population aged 60 and older suffer from hypertension [7]. Effective prevention strategies are urgently needed to prevent hypertension-related and life threatening consequences, particularly heart attack and stroke.

Antihypertensive therapy is highly recommended and has been widely adapted as a standard measure for secondary prevention of hypertension, including prevention of heart attack and stroke [8–10]. Clinical trials have documented the beneficial effects of antihypertensive medication in reducing the risk of cardio- and cerebrovascular morbidity and mortality [8,11,12]. Compared to placebos, receipt of antihypertensive therapy is associated with 35–40% reductions in the incidence of stroke, 20–25% reductions in heart attack, and 50% reduction in heart failure [13]. Despite the substantial preventive effect demonstrated through the randomized controlled trials described above, the majority of patients on antihypertensive medication remain at high risk for sever cardiovascular complications, underscoring the need for more innovative measures for secondary prevention.

Lifestyle modification has long been recognized as an effective measure for both primary and secondary prevention of hypertension [8,12,14,15]. Among various lifestyle factors, great attention has been paid to physical activity. The relationship between physical activity and hypertension has been well established [16–18]. Evidence-based interventions are also reported, and are made available for use to promote physical activities [14,19–21]. A recent meta-analysis of nine randomized controlled trials [22] indicated that compared to control conditions, receipt of a physical activity intervention can reduce systolic blood pressure up to 5–10 mmHg and diastolic 1–6 mmHg, respectively. Despite the promising results, there are limitations to these studies. Most of them target young people and the sample size is often small (typically 20–30 participants per arm). Furthermore, none of these trials has assessed hypertension-related life threatening events, such as heart attack and stroke.

It is challenging to motivate people to participate in physical activity in general population [19,22,23]. The challenge is much greater to foster physical activities among old adults who also suffer from hypertension. Hypertensive patients are often old in age with rather diverse backgrounds due to their different and long-term experiences of life [24,25]. In addition to the disease, many other intra-personal and social factors may prevent hypertensive patients from engaging in regular physical activities, such as less education, low income, misperceptions of being old, lack of self-confidence, and isolation from family and the society [3,25]. An effective intervention must overcome these obstacles.

A contemporary approach to address challenges for physical activities is to apply valid theories as guidance [22,23,26], a typical example is the transtheoretical model [27]. In this model, the process of physical activity is framed into five gradual and progression stages against which physical activity is assessed, goals are set and progressions are monitored. This model also emphasizes knowledge, self-efficacy, decision making, and self-motivation in promoting behavior change. Transtheortical model-based interventions are often reported in the literature [19,28], including studies conducted among senior Chinese adults to foster physical activity [24]. The transtheoretical model would be more promising if it is extended to include other models such as *model of personalized medicine* [29,30] for tailored intervention and *social capital theory* [31,32] to deal with isolation, low self-confidence and lack of social support. However, no reported study has ever tested any of this type of extended models.

Prevention of heart attack and stroke among hypertensive patients is a top priority for chronic disease prevention and control in Wuhan where 50.8% of the population aged 55 years and older are diagnosed with hypertension [33]. With governmental support, Wuhan Center for Disease Prevention and Control (Wuhan CDC) launched the Community-Based Hypertension Control Program (CBHCP) in 2006. The program now covers 114 community health centers (CHC) spread in all over its seven urban districts with an enrollment of 336,958 patients. Following the guideline recommended by the Chinese Medical Association [10], community residents diagnosed with hypertension are managed through the CBHCP at the local CHCs to receive the standard care, including antihypertensive treatment using recommended drugs (e.g., Nefedipine, Amlodipine besylate, Reserpine, and Felodipine) and periodic physical checkup and counseling. We feel an urgent need to include lifestyle changes, particularly physical activity to further improve the preventive effect of CBHCP.

To meet the prevention need, we developed the program *Keep Moving toward Healthy Heart & Healthy Brain* (KM2H^2^) to encourage physical activities among CBHCP patients. Pilot-test among 105 patients indicated that KM2H^2^ was well accepted and appeared to enhance physical activities in hypertensive patients [34]. The purpose of this study is to test the efficacy of the intervention program over control among senior hypertensive patients who are receiving the standard community-based prevention care. Intervention efficacy is measured primarily as reductions in the incidence of stroke and heart attack; separately, secondarily as reductions in the blood pressure, and thirdly as increases in the level of physical activity.

## Materials and Methods

### Ethics statement

The study protocol was approved by the corresponding Institutional Review Boards at Wuhan CDC, Hong Kong Polytechnic University, China, and the University of Florida, USA. All participants signed the informed consent.

### Participants

Participants were hypertensive patients enrolled in and followed up by the CBHCP of Wuhan from April 20, 2011 to November 28, 2011. Patients who met the following five inclusion criteria were included: (1) 55years of age or older, (2) diagnosed with hypertension by a doctor or a physician, following the standard procedure [10], (3) currently on antihypertensive medication, (4) be able to attend intervention research activities, including lectures, group-meetings, and telephone counseling, and (5) can safely participate in moderate or higher levels of physical activities. We set the age limit for this project because the legal retirement age in China is 55 and retired patients were more accessible and had adequate time to participate in research studies.

Participants were excluded if they met the following exclusion criteria: (1) failing the Physical Activity Readiness Questionnaire test (American College of Sports Medicine, 2010; responded positively to any six of the 7 test questions except the use of anti-hypertensive medication); (2) unable to complete the research study due to physical and mental abnormalities; (3) suffering from medical conditions (e.g., dementia, sick in bed due to stoke or heart attack) that may interfere with participating in intervention and/or engaging in required levels of physical activities; and (4) electrocardiogram evidence of heart dysfunction.

### The intervention program KM2H2

The program KM2H^2^ was developed through a team effort led by Dr. Gong and established through rigorous pilot tests. A progressive strategy was used to devised the program, guided by the *transtheoretical model*[19,27] with extension to include the *model of personalized medicine* [29,30] and the *social capital theory*,[31,32] and supported by data from our preliminary studies [35] and others [19,36]. A draft program was first developed by a group of experts in public health and health education from Wuhan CDC, Hong Kong Polytechnic University in China, Wayne State University in the United States, and World Health Organization Collaboration Center for Community Health Services. Others involved in program development included physicians and nurses at various CHCs, community leaders, and retired officials. A number of hypertensive patient volunteers and their family members were also involved. The draft program was circulated among the key investigators of the study for feedback and revision to produce a pilot version. The pilot version was tested among 105 patients to establish the procedure and to obtain feedback for further revision [34].

KM2H^2^ consists of six intervention sessions and two booster sessions. The six intervention sessions include two lectures, two sessions of telephone counseling, and two group meetings, all delivered on a weekly basis. *Session I*, “Understanding High Blood Pressure”, 45–60 minutes, is devised as a group lecture. It aims at motivating participants to engaging in physical activities by provision of basic knowledge about hypertension, risk and protective factors, and blood pressure self-management. This session is delivered through lecturing, demonstration and participation at the community settings.

*Session II*, “The Secrets for Blood Pressure Control through Exercise”, is also a lecture session lasting for 45–60 minutes. This session is devised to empower the participants to engage in physical activity with focus on skills training. The main contents include (1) understanding the concept of “regular and moderate physical activity”, (2) identifying various types of activities that are commonly practiced by people in the community that can be *converted* into regular and moderate physical activity and, (3) learning methods and skills for achieving regular and moderate level of physical activity, including self-scheduling, self-assessment, and log-keeping, and (4) safety measures during exercise.

*Session III*, “Individualized Physical Activity Counseling” is devised for telephone counseling, and it lasts for approximately 10–20 minutes per person. Guided by the model of personalized medicine [29,30], the purpose is to help remove obstacles with which a participant confronted while engaging in physical activity after the first two intervention sessions. Trained interventionists call individual participants at their home to initiate the process. After checking with a patient about his or her blood pressure, medication, and other health conditions, the interventionists focus mainly on physical activity, including participants’ understanding of the meaning of regular and moderate physical activity, self-assessment of physical activity stages, plans to move to the subsequent stages, and obstacles preventing engagement in physical activities. Data from baseline survey was also used as an information source supporting the tailoring of the telephone counseling. To ensure intervention fidelity, (1) all telephone counseling must be tape-recorded; (2) one fifth of the recordings are randomly selected for assessment; and (3) the Consultation Manual was strictly followed as guidance to the telephone counseling at all times.

*Session IV*: “Experience Exchange for Successful Exercise”, is devised as a group session to further enhance the effect from the previous three sessions. Coordinated by two interventionists, the session typically includes 8–10 participants meeting together as a group and it lasts 45–60 minutes. Guided by the social capital theory [31,32], groups are initiated and organized by participants themselves with the assistance of an interventionist. The purpose is to provides opportunities for the participants to associate with each other, to share skills, experiences, and lessons, to assist each other in problem solving, and to build durable friendships for social support [37].

*Session V* is a second one-on-one telephone counseling as described in Session III. The purpose is to help remove new obstacles. Likewise, *Session VI* is another group meeting as in Session IV to strengthen the intervention effect.

**Fig 1:**
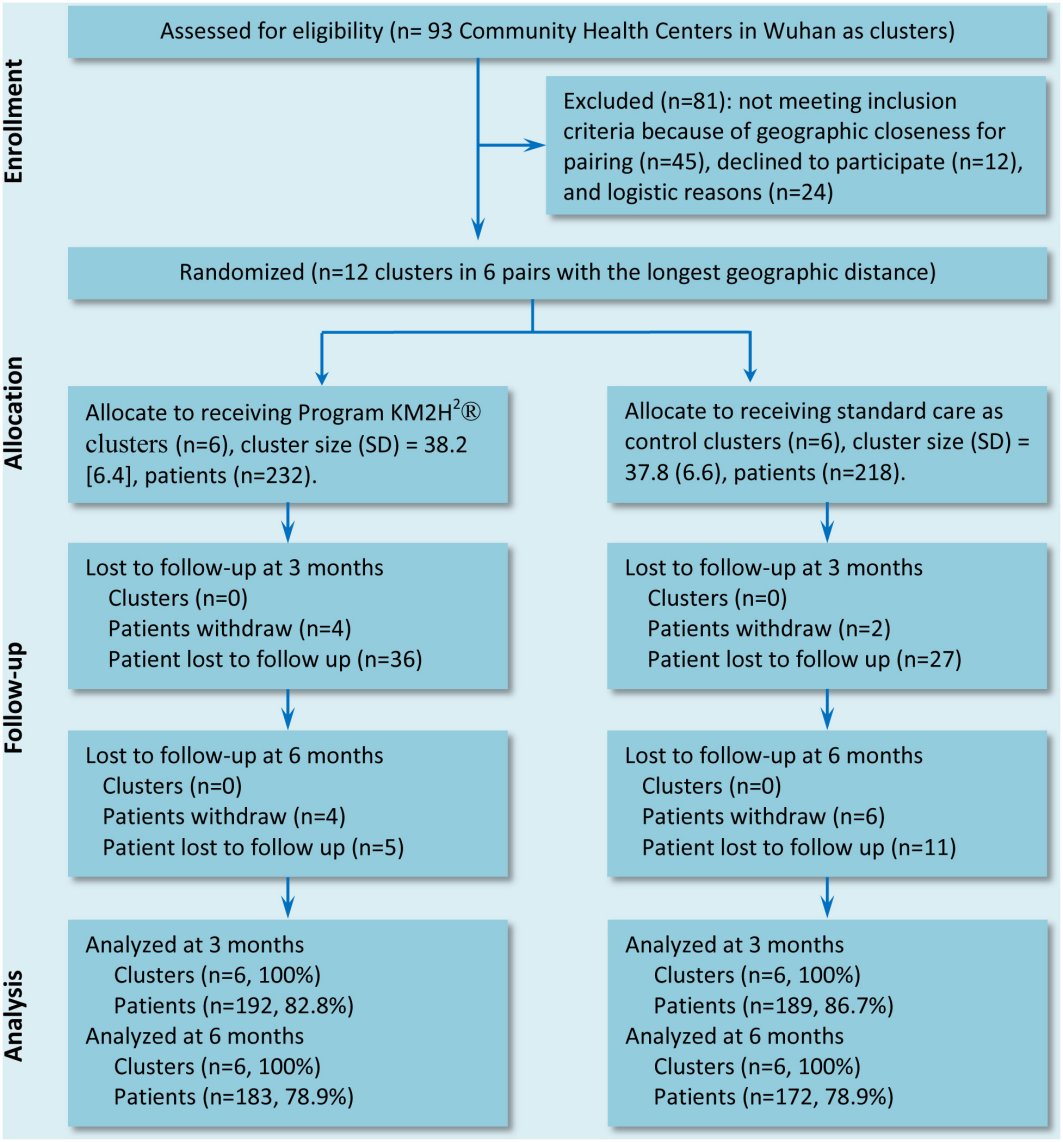
Study Design (CONSORT Flow Diagram).

After the 3-month follow-up assessment, a two-session *booster* is arranged starting with a telephone-counseling, followed by a group meeting. Fig 1 summarizes the time schedule for program delivery, including the six intervention and the two booster sessions.

The intervention was delivered by 35 trained interventionists, five were doctors in preventive medicine from Wuhan CDC and the rest were CHC physicians. The two lecture sessions were delivered by doctors from Wuhan CDC, while the rest were delivered by other interventionists. The interventionists all received one-week fulltime training. On the fifth day of training, all trainees were required to take part in the qualification exam. Additional one-day training for telephone counseling was provided for trainees who were assigned to provide the counseling intervention. Trainees who participate in both trainings and passed the two exams were certified as interventionists to deliver the KM2H^2^. For two CHS physicians who did not pass, additional training was provided until they passed the exams.

### The Standard CBHCP care as control conditions

The standard CBHCP care includes anti-hypertensive medication and periodic follow-up assessments and counseling at the community health center or patients’ home, and patients initiated assessments. The care includes periodic monitoring of blood pressure and hypertension related cardiovascular and other complications, medication adherence, general physical checkup, and psychological counseling.

### Study design

A two-arm longitudinal cluster randomized controlled trial design was used to assessed the efficacy of KM2H^2^. Subjects were recruited from 12 community health centers located in six urban districts. Among all the community health centers in a district, the two with longest geographic distance were selected and randomized into either to receive KM2H^2^ or the standard care. The purpose of this arrangement was to avoid potential intervention contaminations. Considering refusal and lost to follow-up, approximately 50 participants per center were approached with an expected sample size of 450 patients at the last follow-up assessment 6 months post-intervention. The sample size was determined through statistical power analysis considering the number and size of cluster and three-wave longitudinal data collection. In the power analysis, type I error was set at *α* < .05 and type II error at *β* < .02 to detect a moderate effect size.

Patients were contacted with the assistance of the participating community health centers. Among the total 586 participants approached, 77% were eligible according to the inclusion and exclusion criteria. Of the eligible patients, 450 signed the informed consent form were included in the study. These participants were randomly assigned, by center, to receive either KM2H^2^ (n = 232) plus the standard CBHCP care or the standard CBHCP care only (n = 218). The differences in the number of subjects between the two groups were due to the group-based randomization method with unequal number of patients in different community health centers. The CONSORT diagram (Fig 1) illustrates the study design, including sampling, randomization, intervention and assessments.

### Procedure of data collection

Data were collected using the Community-Based Hypertension Self-Management Questionnaire we developed and pilot-tested for this project. The questionnaire was completed through in-person interview by trained data collectors and it took approximately 40 minutes on average to complete. Three waves of data were collected, including baseline, the first and the second follow up at 3 and 6 months respectively. Reminding calls were made before a scheduled date of data collection. For participants who did not show up on the scheduled day, at least one follow-up schedule was attempted. Data collectors were 10 senior-year public health graduate students who were on practical training at Wuhan CDC. These data collectors received one-day training before they were dispatched to interview patients with standard data collection protocol. The data collectors were all blinded to the intervention conditions.

Participants were compensated with a free meal (~3 US dollars). They were also offered free physical exam, electrocardiogram and lung function test upon the completion of each wave of data collection. The KM2H^2^ was well accepted and no complaints about the intervention were reported by any of these participants during the course of intervention. The documented reasons for lost to follow-up (1) at 3 months were moving out (n = 5), lost contact (n = 5), hospitalized (n = 1), and absent (n = 58); and (2) at 6 months were moving out (n = 10), lost contact (n = 6), sick (n = 1), and absent (9).

### Data availability

All data used for this study are available free of charge by contacting Dr. Jie Gong or Dr. Xinguang Chen.

### Variables and their measurement

To address the purpose of this study, the primary, secondary and tertiary outcome variables measured at all three waves of data collection were used.

#### Heart attack and stroke

These two cardio- and cerebrovascular events were used as the primary outcome. Since all participants were recruited from CBHCP, they were knowledgeable about heart attack and stroke through periodical visits to the community health centers for care. This greatly facilitated our effort to count the cardio- and cerebrovascular events for program effect evaluation. Only new events of heart attack and stroke during the follow-up were included in analysis; and heart attack and stroke were assessed separately. To maximize the likelihood to find new events, all participants were asked, at each follow-up assessment, the question, “Please think back the past three months since last interview on [date of the interview]. During this period, have you suffered from or diagnosed by your doctor as *having a heart attack*?” For patients who responded positively to the questions, they were further questioned regarding number of attacks, symptoms, where and when the attack occurred. To verify each reported events, an investigator was dispatched to CHC to contact the physician in charge of that patient to review the medical record and to discuss with the doctor to determine the diagnosis. An exactly the same approach was used to assess the occurrence of stoke during the two follow-up periods.

#### Blood pressure

Blood pressure (mmHg) at rest was used as the secondary outcome to support the efficacy of KM2H^2^. Both systolic (SBP) and diastolic blood pressure (DBP) were measured with the mercury sphygmomanometers. Following the standard protocol recommended by the American Hypertension Association, participants were asked not smoking, not drinking, no coffee or tea, and not engaging in physical activities at least for 30 minutes before assessment. During the measurement, participants were instructed to sit down and relax at least for 5 minutes before the blood pressure was taken. Blood pressure was measured by registered nurses. Mean values of two reliable measures at least 3 minutes apart were used for analysis.

#### Transition stage toward physically active

This was the first tertiary outcome variable to support the efficacy of KM2H^2^ in encouraging participants moving from physically non-active to more active and further to maintaining active. Participants were asked to self-rate their physical activities using a self-rating scale according to the definition of *moderate to intense activities* (30 minutes of activities a session for at least 3–5 times per week, including brisk walking, jogging, bicycling, swimming, or any other activities with similar intensity). This scale was developed for use in research to assess physical activities among senior hypertensive patients with the guidance of the transtheoretical model [35]. It consists of five questions, corresponding to the five transition stages with 1 = *pre-contemplation stage* (no intention to engage in the physical activity in the next 6 months), 2 = *contemplation stage* (would like to engage in the physical activity in the next 6 months), 3 = *preparation stage* (started to engage in), 4 = *action stage* (engage in the physical activity but have not done it for 6 months), and 5 = *maintenance stage* (continue physical activities for more than 6 months).

#### Levels of physical activity

This is the second tertiary variable regarding physical activity. It was assessed by asking participants if they were currently participating in a list of physical activities (y/n), including brisk walk, tai chi, jogging, bicycling, swimming, sweeping floor, and house cleaning. This list was created following the recommend definition of regular physical activity (RPA)[36] and through rigorous pilot testing. For those who reported participating in any of the listed physical activities, they were further prompted to report the number of times they have engaged in the activity in the past week, the average duration (in minutes) of an session of exercises on a typical day using a 4-point rating scale with 0 = *no exercise*, 1 = *less than 15 minutes*, 2 = *15–29 minutes*, 3 = *30–59 minutes*, and 4 = *1 hour or more*. Participants who were not currently engaging in physically activities were automatically scored zero as *no exercise*. This measure has been validated and is widely used in survey studies to assess levels of physical activities in both etiological [35,38] and intervention studies [39].

#### Covariates

Variables included as covariates were: Demographic, including age (in years), gender (male/female), monthly income (RMB, <1000, 1000-, and ≥2500); behavioral, including smoking (yes/no), drinking (yes/no); years of hypertension (<5, 5-, 15- and ≥20); family history (y/n) of hypertension, diabetes, stroke, heart disease, and hyperlipidemia; and self-reported adherence to antihypertensive medication (no self-reduction, no skip, and no self-stop). These variables were used for descriptive statistical analysis to characterize our study sample and some of them were used in multivariate models to evaluate the intervention effect.

### Statistical analysis

The intention to treat (ITT) approach was used for evaluation. Baseline comparability and attrition rates of the two intervention arms were assessed first before evaluation. Baseline comparability assessment is needed because we used a cluster randomized design which does not always result in comparable groups. Chi-square test (for categorical variables) and student t-test (for continuous variables) were used to assess baseline comparability of the outcome variables before the evaluation of the intervention program.

The program efficacy in increasing physical activity, reducing blood pressure and risk of heart attack and stroke was assessed at 3-month and 6-month post-intervention respectively. The student t-test was used to assess the difference in the behavioral measures (scale scores) and chi-square test was used to compare differences in the incidence rates of heart attack and stroke between the intervention and the control groups, followed by effect size (Cohen’s *d*) calculation with the criteria *d* = 0.2 as small effect, 0.5 as medium effect and 0.8 as large effect [40,41].

Results from the comparative analyses described above regarding the association between receiving KM2H^2^ and the outcome variables, relative to the standard care group were further verified using multivariate models. The mixed effect modeling method (for continuous measures such as levels of physical activity, SBP and DBP) and the generalized mixed effect modeling method (for binary variables such as the events of heart attack and stroke) were used for the verification analysis. The mixed effect modeling approach was used to adjust for the design effect of the cluster-randomization, repeated measurements of three waves of longitudinal data, potential confounding from baseline differences in the outcome variables, and key covariates, and to handle missing data at follow-ups [42].

A total of six models were constructed and used in the analysis, including two models for the primary outcome measures (heart attack and stroke), two for the secondary outcome measures (SBP and DBP) and two for the tertiary outcome measures (tendency toward and levels of physical activity). After allowing for the random intercept and random slope and the fixed effect of time and intervention groups, the *interaction* between time and intervention conditions (fixed effect) was used as evidence to support the efficacy of KM2H^2^ on the outcome variable. Instead of presenting the estimated beta coefficients from the mixed modeling analyses, we reported the differences between the intervention and the control in blood pressure and incidence of stroke and heart attack for easy understanding and reviewing without sophistically training in biostatistics. In conducting the referential statistical analyses, *p* < .05 (two-tailed) was used as the criterion. Statistical analyses were completed using the software SAS version 9.30 (SAS Institute, Inc., Cary, NC).

### Registration

The study was officially registered in ISRCTN (http://www.isrctn.com/), and the trial registration number is ISRCTN12608966.

## Results

### The sample and their baseline comparability

**Fig 2:**
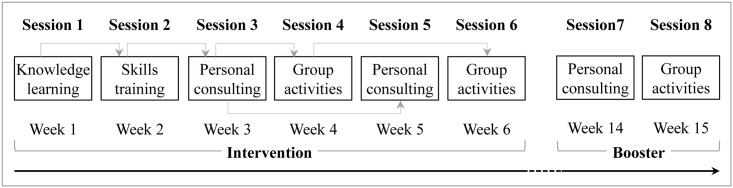
Scheduling of Program Delivery for the KM2H^2^.

Among the total 450 participants, 381 (85%) were followed up at three months and 355(79%) at six months post-intervention. The follow-up rates were not statistically significant different between the two intervention arms (Fig 2 the Consort Chart and Table 1). In addition to the variables at baseline, we also compared the variable adherence to anti-hypertension medication at the 3- and 6-months follow-up, and found no significant differences between the KM2H^2^ and the standard care group.

**Table 1:** Comparison of the baseline characteristics of the study participants in the KM2H^2^ (intervention) group and the Standard Care (control) group.

Variables	Intervention (N = 232)	Control (N = 218)	Overall (N = 450)	*Difference np* value
**Gender, % (n)**				
Male	43.1 (100)	40.8 (89)	42.0 (189)	
female	56.9 (132)	59.2 (129)	58.0 (261)	0.625
**Age in years, % (n)**				
55–59 years	22.8 (53)	30.3 (66)	26.4 (119)	
60–64 years	32.8 (76)	27.5 (60)	30.2 (136)	
65–69 years	28.4 (66)	20.6 (45)	24.7 (111)	
70+ years	16.0 (37)	21.6 (47)	18.7 (84)	
Mean (*SD*)	64.3 (5.6)	64.0 (6.5)	64.2 (6.1)	0.662
**Family income, % (n)**				
<1000 RMB	9.3 (42)	23.8 (52)	20.9 (94)	0.001
1000–2499	47.8 (111)	56.4 (123)	52.0 (234)	
2500+	33.2 (77)	17.4 (38)	25.6 (115)	
Missing	0.9 (2)	2.3 (5)	0.9 (7)	
**Marital status**				
Married/cohabit	83.5 (216)	93.1 (182)	88.4 (398)	0.005
Divorced/separate	4.3 (10)	11.9 (26)	8.0 (36)	
Missing	2.6 (6)	4.6 (10)	3.6 (16)	
**Habital smoking, % (n)**				
Yes	13.8 (32)	24.3 (53)	18.9 (85)	0.004
**Habitual drinking, % (n)**				
Yes	17.7 (41)	17.9 (39)	17.8 (80)	0.952
**Blood pressure (mmHg)**				
Systolic, mean (SD)	140.7 (17.1)	141.7 (17.3)	141.2 (17.2)	0.565
Diastolic, mean (SD)	86.1(9.3)	87.0 (12.3)	86.5 (10.8)	0.399
**Years of hypertension, % (n)**				
<5 years	26.3(61)	37.6(82)	31.8 (143)	0.021
5–14 years	42.2(98)	39.5(86)	40.9 (184)	
≥15 years	31.5(73)	22.9(50)	27.3(123)	
Mean (SD) years	13.0(10.5)	12.0(10.9)	12.5(10.7)	0.341
**Family history**				
Hypertension	60.8(141)	56.4(123)	58.7(264)	0.349
Diabetes	10.8(25)	10.6(23)	10.7(48)	0.938
Stroke	17.7(41)	12.4(27)	15.1(68)	0.118
Heart diseases	13.8(32)	9.2(4.4)	11.6(52)	0.126
Hyperlipedemia	6.9(16)	8.3(18)	7.6(34)	0.585
**Medication adherence, %(n)**	69.4 (161)	70.2 (153)	69.8 (314)	0.856

### Effect size on physical activity

Results from mixed effect modeling analysis (the upper panel of Table 2) indicate that relative to the control condition, receiving KM2H^2^ was associated with higher physical activity stages at three months (Cohen’s *d* = 0.64, 95% CI: [.35, .93]) and six months (Cohen’s *d* = 0.75, 95% CI: [.42, 1.09]) post-intervention respectively. The actual physical activity levels (the lower panel of Table 2) were also significantly higher for the KM2H^2^ participants than for the control participants at three (*d* = 0.53, 95% CI: [0.21, 0.85]) and six months (*d* = 0.45, 95% CI: [.04, 0.85]) post-intervention. In addition, the effect appeared to be greater for tendency toward than the actual level of physical activity, and males appeared to do better than females in these measures.

**Table 2:** Program effect (differences and effect size) on tendency toward physically active and actual levels of physical activity between the KM2H^2^ (intervention) and the Standard Care (control) group, overall and by gender.

Time/variables	Intervention Mean (SD)	Control Mean (SD)	Adjusted Diff. (SD)	Effect Size(ES)	95% CI of ES
**Tendency toward physically active**					
*Total sample*					
Baseline	3.54(1.62)	3.27(1.67)	0.00 (n/a)	n/a	n/a
3 Months	3.95(1.27)	2.96(1.59)	0.88(1.38)	0.64***	0.35, 0.93
6 Months	4.35(1.16)	3.26(1.62)	1.01(1.34)	0.75***	0.42, 1.09
*Male*					
Baseline	3.80(1.54)*	3.30(1.66)	0.00 (n/a)	n/a	n/a
3 Months	4.01(1.25)	2.86(1.52)	1.09(1.31)	0.83**	0.38, 1.27
6 Months	4.37(1.18)	3.17(1.57)	1.13(1.31)	0.86***	0.46, 1.27
*Female*					
Baseline	3.36(1.67)	3.25(1.68)	0.00 (n/a)	n/a	n/a
3 Months	3.90(1.29)	3.02(1.64)	0.77(1.43)	0.54**	0.19, 0.88
6 Months	4.32(1.16)	3.32(1.65)	0.93(1.35)	0.69**	0.25, 1.12
**Levels of physical activity**					
*Total sample*					
Baseline	2.73(1.54)	2.68(1.40)	0.00 (n/a)	n/a	n/a
3 Months	2.81(1.35)	2.14(1.74)	0.74 (1.40)	0.53**	0.21,0.85
6 Months	3.37(1.28)	2.66(1.81)	0.54 (1.22)	0.45*	0.04,0.85
*Male*					
Baseline	2.79(1.48)	2.38(1.48)	0.00 (n/a)	n/a	n/a
3 Months	2.83(1.27)	1.83(1.76)	1.16(1.32)	0.87*	0.21, 1.54
6 Months	3.53(1.08)	2.41(1.87)	0.49(0.99)	0.49	-0.70, 1.68
*Female*					
Baseline	2.68(1.59)	2.91(1.31)	0.00 (n/a)	n/a	n/a
3 Months	2.80(1.41)	2.33(1.71)	0.44(1.41)	0.31	-0.12, 0.74
6 Months	3.25(1.40)	2.80(1.77)	0.47(1.26)	0.37	-0.07, 0.82

**Note**: The program effect for each outcome variables was estimated using a Mixed Effect model to adjust for the cluster randomization, baseline difference, repeated measurement, and covariates. SD: Standard deviation; CI: Confidence interval; n/a: not applicable. Adjusted diff.: Adjusted differences,

*: *p* between 0.05–0.01,

**: *p* between 0.01–0.001, and

***: *p* < 0.001.

### Reductions in blood pressure

Results from mixed effect modeling analysis in Table 3 indicate that relative to the standard care control conditions, receiving KM2H^2^ was associated with 3.72 (SD = 14.33, effect size *d* = -0.26) mmHg reductions in SBP and 2.92 (SD = 8.14, effect size *d* = -0.36) mmHg reduction in DBP at 6 months follow-up.

**Table 3:** Changes in blood pressure after receiving the intervention KM2H^2^, compared to the controls with standard care, results from the mixed effect modeling analysis, by gender.

Time/variables	Intervention Mean (SD)	Control Mean (SD)	Adjusted Diff. (SD)	Effect Size ES	95% CI of ES
**Systolic blood pressure**					
*Total sample*					
Baseline	140.72 (17.14)	141.67 (17.30)	0.00 (n/a)	n/a	n/a
3 Months	140.06 (14.93)	140.57 (16.43)	-0.71 (14.73)	-0.05	-0.36, 0.26
6 Months	136.15 (16.00)	138.38 (15.31)	-3.72(14.33)	-0.26	-0.68, 0.16
*Male*					
Baseline	140.86 (16.74)	142.78 (16.86)	0.00 (n/a)	n/a	n/a
3 Months	142.26 (13.71)	142.75 (16.60)	-2.19 (14.28)	-0.15	-0.71, 0.41
6 Months	137.62 (16.43)	137.44 (16.22)	-2.46 (14.69)	-0.17	-0.79, 0.45
*Female*					
Baseline	140.61 (17.51)	140.93 (17.61)	0.00 (n/a)	n/a	n/a
3 Months	139.09 (15.73)	139.23 (16.26)	0.14 (15.04)	0.01	-0.34, 0.36
6 Months	135.22 (15.76)	138.97 (14.77)	-4.21 (14.10)	-0.30	-0.78, 0.18
**Diastolic blood pressure**					
*Total sample*					
Baseline	86.11 (9.32)	86.99 (12.26)	0.00 (n/a)	n/a	n/a
3 Months	85.52 (9.55)	87.23 (11.32)	-1.30(9.34)	-0.14	-0.61, 0.33
6 Months	81.19 (9.17)	84.50 (10.03)	-2.92 (8.14)	-0.36	-0.99, 0.27
*Male*					
Baseline	86.80 (9.17)	88.89 (14.33)	0.00 (n/a)	n/a	n/a
3 Months	87.47 (8.69)	89.63 (11.69)	-1.73 (8.81)	-0.20	-0.84, 0.45
6 Months	80.77 (9.96)	85.44 (10.31)	-3.11 (7.94)	-0.39	-1.39, 0.61
*Female*					
Baseline	85.57 (9.44)	85.72 (10.53)	0.00 (n/a)	n/a	n/a
3 Months	84.04 (9.94)	85.75 (10.88)	-1.30 (9.82)	-0.13	-0.59, 0.32
6 Months	81.46 (8.69)	83.91 (9.85)	-3.43 (8.15)	-0.42	-0.99, 0.15

**Note**: The program effect on blood pressure reduction was estimated using a Mixed Effect model to adjust for the cluster randomization, baseline difference, repeated measurement, and covariates (i.e., age, income, marital status, years of HBP, adherence to medication, smoking and drinking). SD: Standard deviation; CI: Confidence interval; n/a: not applicable. Adjusted diff.: Adjusted differences.

### Effect in preventing heart attack and stroke

**Fig 3:**
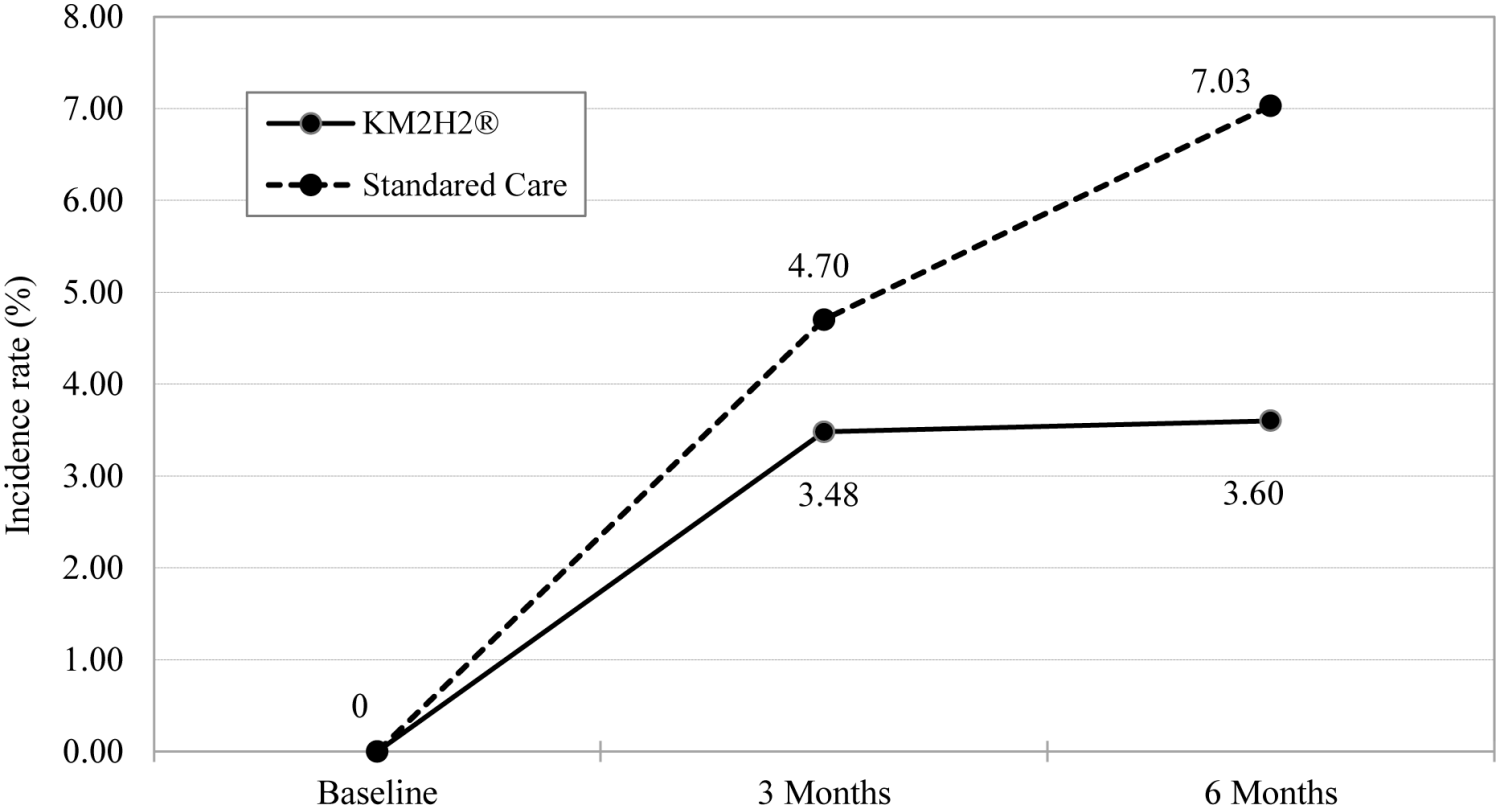
Differences in the incidence rate (%) of heart attack between the KM2H^2^ and the Standard Care Group. Note: The generalized mixed effect model was used to compute the adjusted incidence rate. The random intercept and slope and the main effect of time and group were considered. Covariates of age, gender, marital status, income, year of hypertension, adherence to medication, use of alcohol and tobacco were adjusted. The intervention effect (interaction term in the model) was not significant at 3 months (*p* = .331) and significant at 6 months post intervention (*p* = .028).

**Fig 4:**
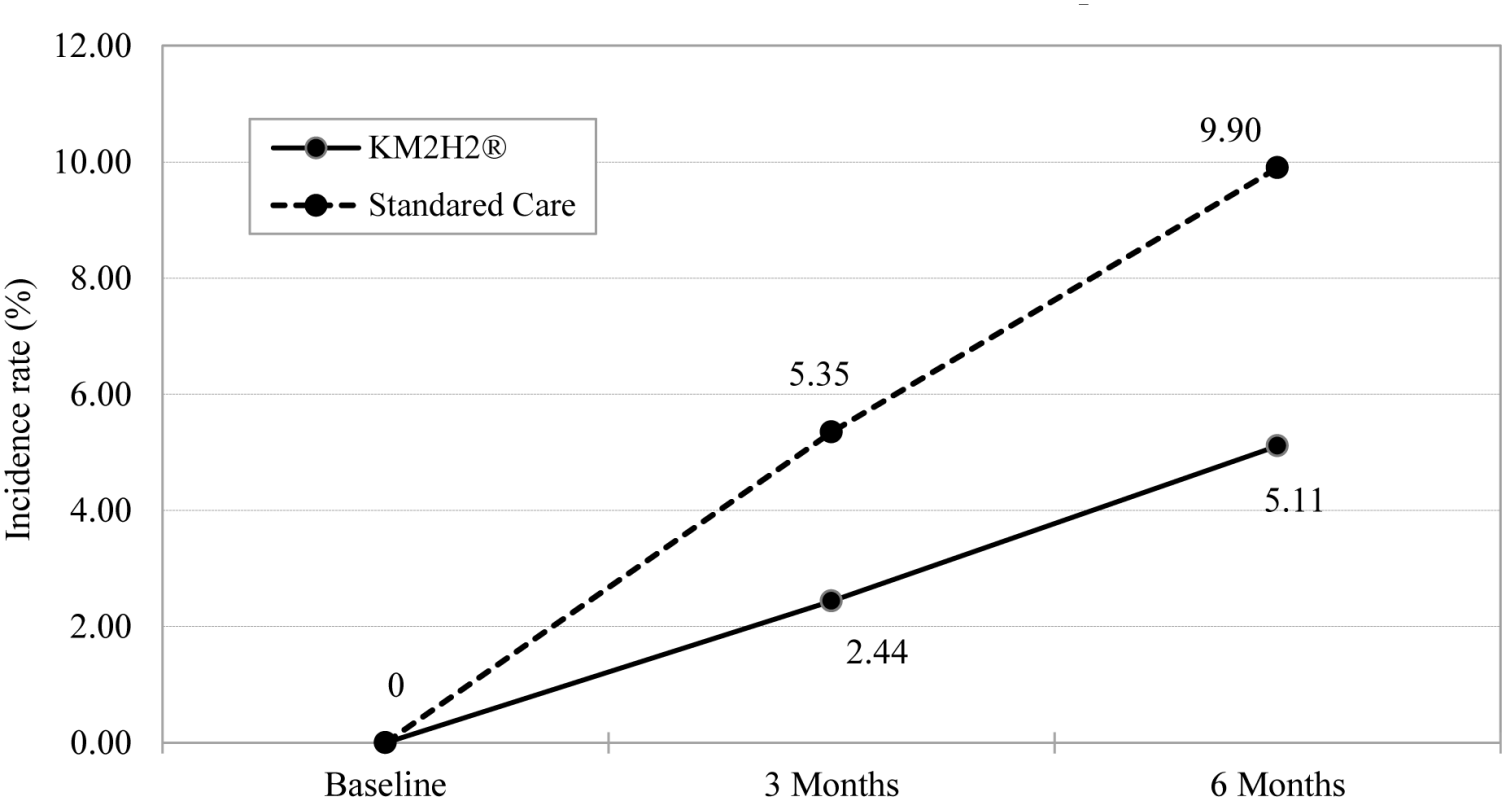
Differences in the incidence rate (%) of stroke between the KM2H^2^ Group and the Standard Care Group. Note: The generalized mixed effect model was used to compute the adjusted incidence rate. The random intercept and slope and the main effect of time and group were considered. Covariates of age, gender, marital status, income, year of hypertension, adherence to medication, use of alcohol and tobacco were adjusted. The intervention effect (interaction term in the model) was not significant at 3 months (*p* = .230) and significant at 6 months post intervention (*p* = .015).

A total of 44 participants (14 from the intervention group and 30 from the control group) were diagnosed with heart attack and 61 with stroke (18 from the intervention group and 43 from the control group) during the 6-month follow-up period. After considering for the design effect of multi-center randomization with repeated measure, the main effect of time and group, the covariates of age, gender, tobacco smoking and alcohol consumption, and the adherence to anti-hypertensive medication, the adjusted incidence rates of stroke and heart attack for the two groups during the follow-up period are presented in Fig 3 and Fig 4. Relative to the standard care at the 6th month post-intervention, receiving KM2H^2^ was associated with significant reductions in the incidence (any events during follow-ups) of heart attack (3.60% vs. 7.03%, *RR* = 0.51, p < .05) with Cohen’s *d* = .37, 95% CI: [.34, .40], and stroke (5.11% vs. 9.90%, *RR* = 0.52, p < .05) with Cohen’s *d* = 0.36, 95% CI: [.32, .40].

## Discussion and Conclusions

In this study, we reported an efficacy trial of the program KM2H^2^ in encouraging physical activity for prevention of heart attack and stroke among a sample of 450 senior hypertensive Chinese patients who are on antihypertension medication. The program was developed with guidance of an *extended transtheoretical model* that incorporates the *model of personalized medicine* and the *social capital theory*. It consisted of two in-house sessions, two telephone-counseling sessions and two group-meeting sessions, all of which were delivered on a weekly basis at the community settings. Physicians in the community health centers comprised the main force of the interventionists to deliver the intervention. The trial was evaluated using the advanced mixed effect modeling approach and three waves of longitudinal data. The effect size of the intervention ranged from medium to large for physical activity measures and medium for the incidence of heart attack and stroke.

The effect of KM2H^2^ was substantial in reducing the risk of heart attack and stroke. Although there is a lack of data on the incidence of stroke and heart attack among senior hypertensive patients in Wuhan, data from population-based studies indicate that among the Chinese adults in general, the annual incidence rate is ranged from 200-600/10000 for stroke (before 2010) [43,44] and 40-60/10000 (during the 1990s) for heart attack [45]. Both stroke and heart attack occur primarily among people living with high blood pressure and approximately half of senior Chinese is hypertensive [33]. Considering a factor of approximately one-fold increases in heart attack and stroke in a decade [44], the estimated incidence rate could be 6–18% for heart attack and 8–24 for stroke among the participants in our study. If the effect of KM2H^2^ can be demonstrated through a formal phase-III trial, large-scale applications of this program will produce substantial effect in reducing the number of heart attack and stroke events among hypertensive patients currently totaling hundreds of millions in China.

The efficacy of KM2H^2^ in reducing the risk of heart attack and stroke was further supported by the impact of the intervention on reduction in blood pressure, the secondary outcome. Relative to the standard care, receiving KM2H^2^ is also associated with significant reductions in both systolic and diastolic blood pressure, supporting the observed reductions in stroke and heart attack. Although the amount of blood pressure reduction was relatively small, the effect was measured over a very short period of six months. Furthermore, the reduction was achieved over the effect of the standard CBHCP care, including antihypertensive drug treatment.

Lastly but not the least is that the effect of KM2H^2^ is supported by the observed increases in physical activity, the tertiary and target outcome variables. Compared to controls, participants in the KM2H^2^ were more likely to move to advanced physical activity stages with higher levels of actual physical activity engagement. These findings provide strong evidence (1) supporting adequate efficacy of KM2H^2^ in encouraging physical activity among senior Chinese hypertensive patients and (2) demonstrating its great potentials for community-based prevention of heart attack and stroke

### Program specificity and relevancy

Why the program KM2H^2^ is efficacious? The first explanation we can offer is *program specificity and relevance*. In preparing for KM2H^2^, we conducted a number of pilot studies to assess the possibilities to encourage physical activity among old adults, including those suffering from hypertension. Results from the pilot studies revealed that many old adults did engage in a number of physical activities by themselves, including quick walk, jogging, running, swimming, tai-chi, and dancing, to name a few, although many of these subjects were in their 60s and several even in their 70s. However, few of these people met the criteria of regular and moderate level activity required for prevention of heart attack and stroke. In these pilot studies, we also identified a number of *barriers* (e.g., lack of knowledge, lack of psychosocial support, and lack of self-confidence) and *facilitators* (e.g., adequate time, some interests, willingness to participate if offered by health professionals and not for commercial purpose) for physical activity [35]. These preliminary data provide first-hand evidence supporting the development of KM2H^2^.

### Theory-based intervention

Theory-ground intervention offers another explanation why KM2H^2^ works. We developed the program following the *extended transtheoretical model*. To motivate senior hypertensive patients to engage in physical activity, we devised the intervention with the first session for knowledge enhancement, considering the low level of education and the lack of knowledge of old hypertensive patients. Physical activities are nothing new at all to many people, including old adults in China; we thus used only one in-house session, Session II for physical activity training with emphasis on skill training and problem-solving. Considering the great individual differences of senior hypertensive patients, two one-on-one counseling sessions were added, guided by the *model of personalized medicine*. To enhance social support and to promote self-confidence, two *social capital theory*-based group sessions were included. These sessions provided opportunities for participants to associate with each other, to share skills and experiences, and ultimately to build a lasting relationship to enhance social capital[31,37] that support the initiation and maintenance of physical activity by increasing access to the enriched social and emotional resources [31,37,46]. The guiding role of the transtheoretical model has been challenged by the lack of observed intervention effect [19]. Our findings suggest the utility of this popular model by inclusion of other related theories and models to ensure upward movement of the intervention recipients along with the physical activity transition stages.

### Limitations and further research

There are several limitations to this study. Although the program was evaluated using a randomized design, a few baseline measures significantly differed between the two intervention arms, including cigarette smoking and marital status, both of which may have confounded the intervention. Although such differences are not uncommon for community-based trials with a cluster randomization design [47], and the potential impact from the differences was carefully handled in this evaluation with advanced statistical methods, efforts should be used in future trials to avoid such bias. For example, in addition to geographic distance, a stratified approach may be considered to ensure more balance design [47,48]. Several variables of the study were measured using self-report with no external validation. Caution is needed when the research findings of this study is applied. The current study can also be improved in several other aspects. For example, in addition to self-report, wireless technologies (e.g., various types of accelerators) can be used to obtain objectively data on physical activity. The attrition rate was relatively high, although those who were lost follow-up did not significantly differ from those retained in the study. More rigorous efforts should be used in future studies to ensure high follow-up rate. Lastly, the primary outcomes heart attack and stroke were measured only for those who reported a cardio- or cerebrovascular events. Participants who experienced an event but did not report were missed. More attention should be paid to this issue in future studies. Despite these limitations, findings of this study provide adequate data supporting a formal evaluation of the effectiveness of KM2H^2^ through more rigorous phase-III trials.

## Supporting Information

S1 CONSORT Checklist CONSORT Extension (referred to Fig 1). (DOCX)

S1 Protocol Study protocol. (PDF)

## Abbreviations

CBHCP: Community-Based Hypertension Control Program
CDC: Center for Disease Prevention and Control
CHC: Community health centers
DBP: Diastolic blood pressure
ITT: Intention to treat
KM2H^2^: Keep Moving Toward Healthy Heart & Healthy Brain
SBP: Systolic blood pressure
