# Supplementary material for: Efficacy of a Community-Based Physical Activity Program KM2H2 for Stroke and Heart Attack Prevention among Senior Hypertensive Patients: A Cluster Randomized Controlled Phase-II Trial
Source: PLoS One. 2015 Oct 1;10(10):e0139442. doi: 10.1371/journal.pone.0139442 (PMC4591119; doi:10.1371/journal.pone.0139442)
Supplement: S1 Protocol — (PDF) [file pone.0139442.s002.pdf]

Research Protocal

Title: Keep Moving Toward Healthy Heart and Brain (KM2H2) – A Phase II Randomized Trial

PI: Jie Gong, MD, PhD, Professor and Associate Director, Wuhan CDC

Background:

Among people over 50 years of age, more than a half is suffering from high blood pressure. These hypertensive patients are at increased risk of stroke and heart attack, two most life-threatening complications of high blood pressure. To control high blood pressure for secondary prevention, Wuhan Center for Disease Prevention and Control established the Community-Based Hypertension Control Program (CBHCP) in 2006 to deliver anti-hypertension medication. The preventive effect of physical activities has yet to be investigated.

Hypothesis

Senior hypertensive patients can be motivated to participate in physical activity and moderate level of physical activity has significant effect to reduce life-threatening consequences of hypertension, particularly the incidence of heart attack and stroke. This hypothesis will be tested using the following research design.

Research Design

Multi-center randomized controlled trial

(1) Selection of intervention contents: To ensure high feasibility and sustainability, the research study focuses on the physical activities commonly practiced by old people in China, including jagging, quick walking, slow running, group-dancing, and bicycling.

(2) Involvement of the study participants: The participants of this study (approximately 450-500) were recruited among patients from 12 community health centers enrolled in the CBHCP. The CBHCP is founded by the Chinese government and implemented by Wuhan CDC. The standard care includes periodically physical check-up, antihypertension medication, and psychological counseling. In this study, participants from the 12 health centers were randomly assigned by health centers with those from six centers receive KM2H2 + standard CBHCP care and those from the remaining six centers receive the standard CBHCP care.

Intervention

The intervention program KM2H2 includes six sessions, plus two booster sessions. The first session covers the basic knowledge about hypertension and motivation of physical activity, the second session is for physical activity training, the third session is one-on-one counselling, the fourth session is small group activity. These four sessions are delivered on a weekly basis. Sessions three and four are repeated once to complete the intervention. The intervention

program is based on an extended Transtheoritical Model to incorporate the Model of Personalized Medicine for individualized counselling and the Social Capital Theory for small group activities. Control conditions are the standard community health care.

If agreed to participate in this research study, the participants will attend two lectures, one per week, at community health centers regarding (a) the knowledge of high blood pressure and consequences, (b) risk and protective factors of high blood pressure with emphasis on physical activities as an important approach for prevention, (c) physical activity training, and (d) training for safety assessment and measures to ensure safety while exercising. After the two lecture sessions, participants will be followed twice. The first one, a week after the lecture, is a one-on-on psychological counseling to coaching safer and appropriate physical activities and to solve issues confronted in engaging is physical activities. One week later, the participants are followed up again through a researcher-guided small group session (8-10 patients) to exchange skills and experience in physical activities for blood pressure control and to foster social and emotional support among the participants. The counseling and small group sessions are repeated one month later to end the intervention. Boosters are arranged at three months post-intervention delivered also through one-on-one counseling and small group activities.

Evaluation

During the course of the research study, participants are asked to complete three waves of survey, including a baseline survey conducted before the intervention and two follow-up surveys conducted at 3 and 6 months post-intervention. During each of these surveys, participants are interviewed by trained investigators regarding their medical conditions, physical activities, heart attack and stroke and other information needed for efficacy evaluation.

Primary outcome measures: Levels of physical activities, incidence of heart attack and stroke and blood pressure

Secondary outcome measures: Blood fats and sugar

Statistical analysis

Consider the multi-center randomized design and repeated measurement, mixed effect model will be used to assess the efficacy of KM2H2 following the guidance of intention to treatment. Type I error is set a $p<.05$ (two sided). Significant reductions in the incidence of stroke and heart attack associated with receiving KM2H2 is used as evidence supporting the effect of the intervention.

IRB Approval

This study protocol has been approved by Wuhan CDC’s IRB.

中国湖北 430015 武汉市江汉北路 24 号 电话：8627-85805111 传真：8627-59512790
24# Jianghan N.Road,Wuhan 430015, P.R.China Tel 8627-85805111 Fax. 8627-59512790
